# Assessing Clinical Severity and Prognosis in Adolescents With Anorexia Nervosa and Atypical Anorexia Nervosa Using the Albumin‐Globulin Ratio

**DOI:** 10.1002/erv.70106

**Published:** 2026-04-04

**Authors:** Eylem Şerife Kalkan, Ayşe Bilge Baklaci, Yelda Kiliç, Sinem Akgül, Nilgün Özgül, Melis Pehlivanturk Kizilkan

**Affiliations:** ^1^ Division of Adolescent Medicine Hacettepe University Faculty of Medicine Ankara Türkiye; ^2^ Department of Statistics Hacettepe University Ankara Türkiye

**Keywords:** albumin‐globulin ratio, anorexia nervosa, atypical anorexia nervosa, disease severity, prognosis

## Abstract

**Objective:**

The albumin‐globulin ratio (AGR) is a biochemical marker reflecting nutritional and inflammatory status, with significant prognostic value in chronic conditions. This study examined its association with clinical/biochemical markers in adolescents with anorexia nervosa (AN) and atypical AN (AAN).

**Method:**

This retrospective study analysed data from 294 adolescents (103 AN, 191 AAN) aged 12–18 years, presented at an adolescent medicine clinic between 2016 and 2023. AGR values were calculated and relationship between AGR and markers of both disease severity (rate of weight loss, vital sign instability, amenorrhoea, hospitalisation need) and prognosis (rehospitalisation, menstrual recovery, achievement of target body weight) was evaluated. An ideal AGR cut‐off of 1.84 was identified using receiver operating characteristic curve analysis.

**Results:**

Mean AGR was higher in AN than AAN (1.85 ± 0.28 vs. 1.73 ± 0.31, *p* < 0.001). AGR correlated negatively with body mass index *z*‐score, blood pressure, and heart rate. AGR ≥ 1.84 was associated with amenorrhoea, hospitalisation need at admission, hypotension, bradycardia, hypothermia, and leucopenia. Higher AGR predicted rehospitalisation during follow‐up and longer menstrual resumption duration.

**Conclusion:**

Unlike other chronic conditions, a higher AGR at presentation was associated with both greater medical disease severity and poorer prognosis in adolescents with AN and AAN, possibly due to relatively preserved albumin alongside decreased globulin.

## Highlights

- Adolescents with AN have higher AGR than adolescents with AAN.
- Higher AGR predicts hospitalisation, vital instability, amenorrhoea, rehospitalisation.
- The AGR may aid risk stratification and guide treatment planning in AN/AAN.

## 1 Introduction

Anorexia nervosa (AN) and atypical anorexia nervosa (AAN) are complex eating disorders that particularly affect adolescents, posing significant challenges to both clinical management and long‐term outcomes (Arcelus et al. 2011). Amount, duration and rate of weight loss, menstrual history, vital signs and laboratory data reflecting nutritional and medical status have been used to assess disease severity, which is essential for guiding key treatment strategies, including how to initiate refeeding and how closely to monitor electrolyte levels (Brennan et al. 2023; Garber et al. 2019; Whitelaw et al. 2018). Variability in individual physiological responses, reliance on self‐reported or retrospective information for weight‐based metrics, and the presence of significant medical or psychological compromise even in the absence of extreme weight loss or at normal weights in AAN can limit the reliability of these measures for some cases (Fayyaz et al. 2024; Vo and Golden 2022). While a comprehensive and individualised assessment remains essential, the use of reliable biomarkers that accurately assess disease severity, reflect medical and nutritional status, and predict clinical outcomes can enhance patient evaluation and care (Y.‐K. Wu et al. 2024). There are studies in the literature that emphasise the significance of biochemical markers in assessing the severity and clinical outcomes of eating disorders, particularly AN (Caldiroli et al. 2023; Gaudiani et al. 2014; Y.‐K. Wu et al. 2024).

The albumin‐globulin ratio (AGR) has emerged as a promising biochemical marker with several potential applications. Albumin, a key indicator of nutritional status, frequently declines in malnourished patients, while globulins, including various proteins involved in immune responses, may reflect systemic stress or inflammation (Wen et al. 2025; P. P. Wu et al. 2019). Consequently, AGR offers a composite measure that captures both the nutritional and inflammatory dimensions of patient health, a dual aspect that is particularly relevant in the context of eating disorders. Although research directly focusing on AGR in the context of AN and AAN remains scarce, the prognostic value of AGR has been well established in other chronic conditions. A lower AGR has been consistently associated with poorer outcomes and increased mortality in a variety of conditions, from solid tumours to cardiac, gastrointestinal, liver, and renal diseases (Li et al. 2023, 2021; Niedziela et al. 2018; Salciccia et al. 2022; A. Wang et al. 2023; Y. Wang et al. 2022; Xie et al. 2021; Zeng et al. 2020).

Patients with AN typically exhibit both calorie and protein restriction leading to reduced muscle mass and diminished energy expenditure compared to healthy individuals (Rosa‐Caldwell et al. 2023). Early investigations have reported inconsistent findings regarding serum albumin levels in patients with AN. Mira et al. (1987) reported elevated serum albumin concentrations in patients with eating disorders, including AN. In contrast, Umeki observed lower serum total protein and albumin levels in patients with AN compared with healthy controls (Umeki 1988). A case report described a patient with AN who presented with hypoalbuminemia, which normalised following treatment of a concurrent infection (Krantz et al. 2005). Subsequent studies demonstrated that serum albumin concentrations may remain within the normal range even in cases of severe AN (Narayanan et al. 2009; Winston 2012). A recent study have suggested that normoalbuminemia is common in individuals with AN, with 78.6% of patients having normal albumin levels, while 7.1% had hypoalbuminemia and 14.3% had hyperalbuminemia; an increased risk of mortality was observed in those with hypoalbuminemia (Egedal et al. 2025). Furthermore, research has documented that certain globulin fractions, specifically alpha 1‐, beta‐, and gamma globulins, are significantly lower at admission in patients with AN compared to controls, with levels of alpha 2‐ and beta globulins reaching normal ranges by discharge (Roubalova et al. 2021). These findings highlight the dynamic nature of serum protein alterations during the clinical course of AN and their potential role in monitoring disease progression and recovery when used together. Therefore, AGR may serve as a more sensitive marker in predicting nutritional impairment, clinical severity and disease prognosis in adolescents with restrictive eating disorders.

The relative preservation of albumin levels due to generally maintained hepatic synthesis function, longer half‐life and low‐grade systemic inflammation, coupled with the potential more pronounced reduction of globulin concentrations, mostly immunoglobulins, may result in higher AGR levels especially in AN with greater clinical severity. Taken together, these findings suggest that alterations in serum protein fractions may reflect the physiological consequences of severe energy deficiency in AN; however, the clinical relevance of AGR in adolescents with AN and AAN has not yet been systematically investigated. In this study, we aim to evaluate the relationship between AGR and clinical and biochemical markers in adolescents diagnosed with AN and AAN in order to investigate its utility in predicting disease severity and prognosis. The findings from this study are expected to contribute to improved risk stratification and early intervention strategies.

## 2 Method

### 2.1 Study Population

**FIGURE 1:**
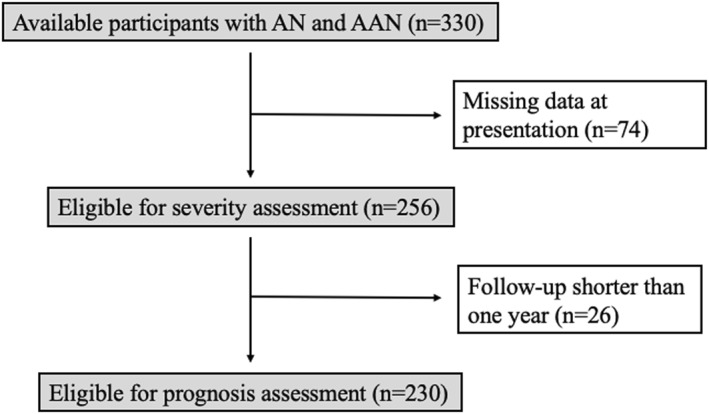
Flow chart for the inclusion of adolescents with AN and AAN.

This retrospective cohort study was conducted among adolescents aged 12–18 years who were diagnosed with AN and AAN according to DSM‐5 diagnostic criteria (American Psychiatric Association 2013) at an adolescent medicine clinic of a tertiary university hospital between June 2016 and May 2023. Individuals with a body mass index (BMI) equal to or higher than 85% of median BMI (mBMI) for age and sex were classified as AAN (Garber et al. 2019). Patients were excluded if they had received any treatment prior to presentation, had an additional chronic disease, signs of active infection, burns, trauma or skin infection that would compromise skin integrity at the time of admission, or any drug use (such as corticosteroids) that would affect albumin and globulin levels. Figure 1 provides a visual representation of how participants were included or excluded from the study. The study protocol was approved by the Health Sciences Research Ethics Committee of Hacettepe University (decision no: 2024/21‐12).

### 2.2 Data Collection and Measurements

All data for the study were obtained from the hospital's electronic medical records and patient files. At this particular clinic, for patients diagnosed with an eating disorder a standardised intake form is routinely completed that captures the following parameters.

#### 2.2.1 Disease Severity Assessment

##### 2.2.1.1 Clinical Parameters

Demographic data such as age and sex and the patients' anthropometric measurements at initial presentation including body weight, height, BMI, mBMI percentage (mBMI%), and BMI *z*‐score were recorded. Body weight and height measurements were obtained from standard measurements taken in the same room and by the same person. Body weight was measured by a digital scale and height was measured by a Harpenden stadiometer. BMI value was obtained by dividing the weight (kg) by the square of the height (m^2^). mBMI% was calculated by dividing the patient’s current BMI by the median BMI (50th percentile BMI for age and sex based on CDC growth charts) and multiplying by 100 (current BMI/mBMI × 100; Centers for Disease Control and Prevention 2024).

The patients’ calorie intake at presentation (kcal), duration of illness (month), duration of weight loss (month), amount of weight lost (kg), minimum and maximum body weights (kg), percentage and rate of weight loss (%), presence and duration of secondary amenorrhoea, and presence of an indication for hospitalisation at admission were scanned. The percentage of weight loss was calculated by dividing the amount of weight loss by the maximum weight before weight loss, and the rate of weight loss was calculated by dividing the percentage of weight loss by the duration of weight loss. The indication for hospitalisation at admission was determined based on the Society for Adolescent Health and Medicine (SAHM) 2022 guideline (Golden et al. 2022).

Vital signs (supine and standing blood pressure, supine and standing heart rate, orthostatic blood pressure and heart rate changes, body temperature) which were measured by the same person in the clinic, and heart rate and QTc interval from electrocardiogram were recorded. Blood pressure measurements (mmHg) were made by using an automated oscillometric device, heart rates (beats per minute) were measured by counting heart rate from the apex for 1 min. Supine measurements were made after 5‐min rest and standing measurements after 3‐min upright. The orthostatic blood pressure change (mmHg) was calculated by subtracting the standing systolic and diastolic blood pressures from the supine systolic and diastolic blood pressures. To find the orthostatic heart rate change (beats per minute), the supine heart rate measurements were subtracted from those recorded while standing. Body temperature (°C) was measured at the external ear using a calibrated digital thermometer. Hypotension was defined as supine systolic blood pressure < 90 mmHg, bradycardia as supine heart rate < 50 beats per minute, and hypothermia as body temperature < 36.0°C. Orthostatic instability was defined as a systolic BP change of > 10 mmHg and a heart rate change of > 20 beats per minute (American Academy of Pediatrics Committee on Adolescence 2003). QTc interval was calculated by dividing the QT interval by the square root of the R–R interval using Bazett’s formula (Bazett 1997).

##### 2.2.1.2 Laboratory Findings

Albumin (g/dL) and globulin (g/dL) values of the patients measured at initial presentation were recorded and AGR was calculated by dividing albumin by globulin. In addition, markers suggesting clinical severity, such as haemoglobin (g/dL), white blood cell (WBC; × 10^3^/µL), absolute neutrophil count (ANC; × 10^3^/µL), absolute lymphocyte count (ALC; × 10^3^/µL), platelet count (× 10^3^/µL), neutrophil‐lymphocyte ratio, platelet‐lymphocyte ratio, alanine aminotransferase (ALT; U/L), aspartate aminotransferase (AST; U/L), total bilirubin (mg/dL), direct bilirubin (mg/dL), blood urea nitrogen (BUN; mg/dL), creatinine (mg/dL), sodium (mEq/L), phosphorus (mg/dL), potassium (mEq/L), calcium (mg/dL), magnesium (mg/dL), glucose (mg/dL), triglyceride (mg/dL), total cholesterol (mg/dL), high density lipoproteins (HDL; mg/dL), low density lipoproteins (LDL; mg/dL), follicle stimulating hormone (FSH; mIU/mL), luteinising hormone (LH; mIU/mL), oestradiol (pg/mL), thyroid stimulating hormone (TSH; mIU/L), free triiodothyronine (T3; pmol/L), and free thyroxine (T4; pmol/L), at admission were recorded. Analyses of biochemical measurements were conducted using standard laboratory methods (Beckman Coulter AU5800 Chemistry Analyser; Beckman Coulter Inc., Brea, CA, USA). Levels of FSH, LH, oestradiol, TSH, T3 and T4 were measured using chemiluminescence immunoassay methods (Immulite 2000 XPi; Siemens Healthcare Diagnostics Inc., Tarrytown, NY, USA). Neutrophil‐lymphocyte ratio was calculated by dividing the neutrophil count by the ALC, platelet‐lymphocyte ratio was calculated by dividing the platelet count by the ALC and BUN‐creatinine ratio was calculated by dividing BUN by the creatinine. The cut‐offs for laboratory data are determined based on general literature data and laboratory reference values (Garber et al. 2019; Sawyer et al. 2016) (Table S1). The optimal threshold for AGR was determined as 1.84 and comparisons for all severity and prognosis data were also made between low and high AGR categories.

Bone mineral density (BMD) measurements requested at the time of presentation were recorded. The assessment of BMD (g/cm^2^) of the lumbar spine (L1‐L4) and femoral head was conducted using dual‐energy X‐ray absorptiometry (DXA) (DXA, Lunar Prodigy Lunar Prodigy Pro; GE Healthcare, Madison, WI, USA). The BMD results were evaluated based on the reference population and expressed as a *z*‐score. According to the recommendations from the guidelines of the International Society for Clinical Densitometry, a BMD *z*‐score ≤ −2 standard deviation (SD) is regarded as ‘below the expected range for age’ (Gordon et al. 2014).

##### 2.2.1.3 Hospitalisation and Refeeding Data

Hospitalisation length (day), the recovery time of the vital signs (day) and the presence of refeeding syndrome were recorded from physician history and hospital's electronic data. Refeeding syndrome was diagnosed based on the criteria established by the recommendations from the SAHM 2022 position paper (Golden et al. 2022; Katzman et al. 2014). Serum phosphorus ≤ 2.9 mg/dL, potassium ≤ 3.5 mEq/L or magnesium ≤ 1.7 mg/dL in the first 7 days after admission were considered as refeeding syndrome.

#### 2.2.2 Prognosis Assessment

Data collected from hospital’s electronic data to assess prognosis include the presence and frequency of additional hospitalisations during follow‐up, presence and duration of the resumption of menstrual cycles, achievement of target body weight (TBW), the time required to reach TBW, the presence of medical remission and the occurrence and frequency of relapses. TBW was defined as 90% of the ideal body weight (IBW) for adolescents with AN. IBW was defined as the 50th percentile weight‐for‐height. For AAN, the previous weight percentile trajectory was identified using historical growth charts, and 90% of that weight percentile was calculated to establish the TBW (Golden et al. 2008; Jary Franklin et al. 2024). Medical remission was defined as reaching and maintaining TBW for at least 3 months, and being recovered in terms of achieving a medical stability. Those with at least 3 months of medical remission followed by a weight loss of more than 10% in 3 months were considered to have relapsed (Golden et al. 2015).

### 2.3 Statistical Analysis

Continuous variables were presented as mean ± standard deviation (SD) and categorical variables were presented as numbers and percentages (%). Normality of continuous variables was assessed using the Shapiro–Wilk test. In continuous variable comparisons between AN and AAN groups, independent samples *t*‐test was used when Shapiro–Wilk test was normal, Mann–Whitney *U* test was used when normality was broken. Categorical variables were compared with Pearson chi‐square test, and Fisher exact chi‐square test when expected cell frequency < 5. Indication for hospitalisation at admission (yes/no) was considered as the most clinically significant short‐term outcome, and the discriminatory power of the AGR for this outcome was assessed using a receiver operating characteristic (ROC) curve. The optimal threshold, which yielded the highest Youden index, was set at 1.84. A sensitivity analysis was performed to assess the stability of the cut‐off point within the range of 1.55–2.01 (Youden range); it was confirmed that the direction/interpretation of the primary outcome did not change within this range. AGR was analysed with two‐sample tests for binary categories (e.g., gender, secondary amenorrhoea) and one‐way ANOVA for variables with three or more categories (e.g., frequency of relapse, number of hospitalisations). The relationships between AGR and continuous variables were given using Pearson correlation when data were normally distributed and Spearman’s rank correlation when normality assumptions were violated. Associations between AGR and categorical variables were evaluated using point‐biserial correlation and two‐sided *p* values. Effect sizes were calculated as Cohen’s *d* for *t*‐tests, rank‐biserial correlation (*r*) for Mann–Whitney *U*, Cramer’s *V* for chi‐square/Fisher tests, eta squared (*η* ^2^) for one‐way ANOVA, and Pearson’s *r* for correlation analyses; interpreted with conventional limits (small = 0.20; medium = 0.50; large ≥ 0.80). All tests were applied as two‐sided, significance level was accepted as *p* < 0.05. Given the large number of pairwise comparisons, *p* values were adjusted for multiple testing using the Benjamini–Hochberg false discovery rate (FDR) procedure. Both raw and adjusted *q* values are reported. Analyses were performed using Python version 3.9.7 (Statistical analyses utilised libraries including pandas (version 2.2.3) and SciPy (version 1.7.1)).

## 3 Results

### 3.1 Demographics and Descriptive Data

Of the 294 adolescents included in the study 103 were AN, 191 were AAN; 261 were female (88.8%), and the mean age was 15.11 ± 1.52 years. Table 1 presents demographic and descriptive data of participants.

**TABLE 1:** Demographics, disease severity and prognosis data of participants.

Demographic characteristic (*n* = 256)	
Age, years, mean ± SD	15.11 ± 1.52
Sex, *n* (%)	
Female	225 (87.9)
Male	31 (12.1)

Disease severity characteristics (*n* = 256)			
Clinical characteristics		Laboratory characteristics	
Body weight, kg, mean ± SD	48.38 ± 11.24	Albumin, g/dL, mean ± SD	4.69 ± 0.37
BMI, kg/m^2^, mean ± SD	18.39 ± 3.51	Globulin, g/dL, mean ± SD	2.70 ± 0.38
mBMI%, mean ± SD	90.91 ± 17.13	AGR, mean ± SD	1.77 ± 0.30
BMI *z*‐score, mean ± SD	−1.02 ± 1.71	Haemoglobin, g/dL, mean ± SD	13.58 ± 1.21
Calorie intake at presentation, kcal, mean ± SD	913.17 ± 407.41	WBC, × 10^3^/µL, mean ± SD	5.98 ± 1.84
Duration of illness, months, mean ± SD	10.09 ± 9.53	ANC, × 10^3^/µL, mean ± SD	3.36 ± 1.49
Duration of weight loss, months, mean ± SD	5.88 ± 5.02	ALC, × 10^3^/µL, mean ± SD	2.07 ± 0.66
Amount of weight loss, kg, mean ± SD	14.10 ± 8.60	Platelet count, × 10^3^/µL, mean ± SD	255.85 ± 61.99
Minimum body weight, kg, mean ± SD	46.12 ± 10.67	Neutrophyl‐lymphocyte ratio, mean ± SD	1.77 ± 1.08
Maximum body weight, kg, mean ± SD	60.79 ± 14.83	Platelet‐lymphocyte ratio, mean ± SD	133.21 ± 45.34
Weight loss percentage, %, mean ± SD	22.51 ± 9.95	ALT, U/L, mean ± SD	17.99 ± 23.43
Weight loss rate, %/months, mean ± SD	6.18 ± 4.93	AST, U/L, mean ± SD	22.01 ± 15.23
Secondary amenorrhoea, *n* (%)	97 (43.3)	Total bilirubin, mg/dL, mean ± SD	0.79 ± 0.53
Secondary amenorrhoea duration, months, mean ± SD	6.24 ± 4.62	Direct bilirubin, mg/dL, mean ± SD	0.15 ± 0.09
Indication for hospitalisation at admission, *n* (%)	48 (18.8)	BUN, mg/dL, mean ± SD	11.40 ± 4.63
Supine systolic BP, mmHg, mean ± SD	98.76 ± 12.56	Creatinine, mg/dL, mean ± SD	0.63 ± 0.12
Supine diastolic BP, mmHg, mean ± SD	60.48 ± 8.25	Sodium, mEq/L, mean ± SD	139.37 ± 2.24
Standing systolic BP, mmHg, mean ± SD	96.27 ± 13.94	Phosphorus, mg/dL, mean ± SD	3.90 ± 0.52
Standing diastolic BP, mmHg, mean ± SD	61.13 ± 10.28	Potassium, mEq/L, mean ± SD	4.30 ± 0.45
Orthostatic systolic BP change, mean ± SD	6.11 ± 5.76	Calcium, mg/dL, mean ± SD	9.97 ± 0.44
Orthostatic diastolic BP change, mmHg, mean ± SD	5.79 ± 5.30	Magnesium, mg/dL, mean ± SD	2.12 ± 0.18
Supine HR, beats/min, mean ± SD	71.83 ± 14.64	Glucose, mg/dL, mean ± SD	79.67 ± 28.06
Standing HR, mean ± SD	90.06 ± 18.82	Triglyceride, mg/dL, mean ± SD	76.34 ± 28.83
Orthostatic HR change, beats/min, mean ± SD	18.81 ± 12.10	Total cholesterol, mg/dL, mean ± SD	190.33 ± 66.64
Body temperature, °C, mean ± SD	36.51 ± 2.06	HDL, mg/dL, mean ± SD	60.37 ± 15.36
Electrocardiogram pulse rate, mean ± SD	67.99 ± 15.73	LDL, mg/dL, mean ± SD	120.34 ± 50.44
Electrocardiogram QTc interval, mean ± SD	0.39 ± 0.03	FSH, mIU/mL, mean ± SD	3.94 ± 2.37
Hospitalisation length, days, mean ± SD	23.04 ± 31.20	LH, mIU/mL, mean ± SD	2.26 ± 3.29
Vital signs recovery time, days, mean ± SD	8.70 ± 7.29	Oestradiol, pg/mL, mean ± SD	31.15 ± 32.04
Presence of refeeding syndrome, *n* (%)	12 (4.8)	TSH, mIU/mL, mean ± SD	1.67 ± 0.96
		fT3, pmol/L, mean ± SD	4.87 ± 0.98
		fT4, pmol/L, mean ± SD	10.59 ± 1.87
		Lumbar BMD *z*‐score, g/m^2,^ mean ± SD	−0.48 ± 1.14
		Femoral head BMD *z*‐score, g/m^2,^ mean ± SD	−0.70 ± 1.21

Prognosis characteristics (*n* = 230)			
Presence of hospitalisation during follow‐up, *n* (%)		Frequency of additional hospitalisations, *n* (%)	
Yes	59 (28.9)	1	52 (88.1)
No	145 (71.1)	≥ 2	7 (11.9)
Resumption of menstrual cycles, *n* (%)	71 (91)	Time required to reach TBW, months, mean ± SD	4.66 ± 5.17
Duration of the resumption of menstrual cycles, months, mean ± SD	7.26 ± 6.58	Presence of remission, *n* (%)	159 (77.9)
Achievement of TBW, *n* (%)	91 (77.8)	Presence of relapse, *n* (%)	84 (38.7)
		Frequency of relapses, mean ± SD	1.79 ± 1.08

Abbreviations: AGR, albumin‐globulin ratio; ALC, absolute lymphocyte count; ALT, alanine aminotransferase; ANC, absolute neutrophil count; AST, aspartate aminotransferase; BMD, bone mineral density; BMI, body mass index; BP, blood pressure; BUN, blood urea nitrogen; FSH, follicle stimulating hormone; fT3, free triiodothyronine; fT4, free thyroxine; HDL, high density lipoproteins; HR, heart rate; LDL, low density lipoproteins; LH, luteinising hormone; mBMI%, median BMI percent; SD, standard deviation; TBW, target body weight; TSH, thyroid stimulating hormone; WBC, white blood cell.

### 3.2 Analysis of AGR Values

The mean AGR for the overall study population was 1.77 ± 0.30. When stratified by eating disorder group, the mean AGR was significantly higher in adolescents with AN (1.85 ± 0.28) compared to those with AAN (1.73 ± 0.31; *p* < 0.001). Table 2 presents the comparison of AGR values according to categorical data. Table 3 presents the relationship between AGR and categorical variables.

**TABLE 2:** Comparison of AGR values across categorical variables with effect sizes.

Characteristics	Category	Total				Anorexia nervosa				Atypical anorexia nervosa			
		AGR	*p*	*q*	Effect size	AGR	*p*	*q*	Effect size	AGR	*p*	*q*	Effect size
General characteristics													
Sex	Female	1.77 ± 0.31	0.185^b^	0.357	0.08^e^	1.85 ± 0.28	0.911^a^	0.911	0.05^d^	1.71 ± 0.31	0.065^b^	0.249	0.14^e^
	Male	1.81 ± 0.29				1.87 ± 0.24				1.80 ± 0.30			
ED‐subtype	AN	1.85 ± 0.28	**<** **0.001** ^b^	0.007	0.25^e^								
	AAN	1.73 ± 0.31											
AN‐subtype	AN‐R	1.78 ± 0.27	0.089^b^	0.240	0.11^e^	1.87 ± 0.28	0.184^a^	0.460	0.42^d^	1.72 ± 0.25	0.778^b^	0.826	0.02^e^
	AN‐BP	1.74 ± 0.39				1.76 ± 0.23				1.73 ± 0.42			
Disease severity													
Secondary amenorrhoea	Yes	1.80 ± 0.33	**0.029** ^b^	0.098	0.13^e^	1.91 ± 0.31	**0.031** ^a^	0.319	0.51^d^	1.65 ± 0.31	0.165^b^	0.474	0.13^e^
	No	1.75 ± 0.29				1.76 ± 0.40				1.73 ± 0.31			
Indication for hospitalisation at admission	Yes	1.86 ± 0.30	**0.005** ^b^	0.023	0.18^e^	1.89 ± 0.30	0.836^b^	0.911	0.02^e^	1.83 ± 0.31	**0.009** ^b^	0.052	0.20^e^
	No	1.75 ± 0.30				1.83 ± 0.27				1.71 ± 0.31			
Presence of refeeding syndrome	Yes	1.88 ± 0.40	0.608^b^	0.702	0.03^e^	1.93 ± 0.41	0.363^b^	0.594	0.31^e^	1.59 ± 0.11	0.417^b^	0.672	0.06^e^
	No	1.77 ± 0.30				1.85 ± 0.26				1.72 ± 0.24			
Prognosis													
Resumption of menstrual cycles	Yes	1.83 ± 0.32	0.370^a^	0.514	0.36^d^	1.91 ± 0.31	0.302^b^	0.594	0.45^e^				
	No	1.71 ± 0.39				1.76 ± 0.40							
Achievement of TBW	Yes	1.78 ± 0.29	0.261^a^	0.440	0.25^d^	1.89 ± 0.27	0.089^a^	0.319	0.43^d^	1.79 ± 0.28	0.826^a^	0.826	0.12^d^
	No	1.85 ± 0.28				1.77 ± 0.29				1.82 ± 0.32			
Presence of hospitalisation during follow‐up	Yes	1.90 ± 0.27	**<** **0.001** ^a^	0.007	0.73^d^	1.94 ± 0.30	0.054^a^	0.319	0.47^d^	1.86 ± 0.24	**<** **0.001** ^a^	0.012	0.85^d^
	No	1.71 ± 0.24				1.81 ± 0.26				1.67 ± 0.22			
Frequency of additional hospitalisations	1	1.84 ± 0.31	0.778^a^	0.778	0.11^d^	1.81 ± 0.26	0.898^a^	0.911	0.07^d^	1.79 ± 0.30	0.688^a^	0.826	0.24^d^
	≥ 2	1.88 ± 0.22				1.94 ± 0.34				1.86 ± 0.06			
Presence of remission	Yes	1.78 ± 0.27	0.184^a^	0.357	0.23^d^	1.89 ± 0.27	0.102^a^	0.319	0.44^d^	1.72 ± 0.25	0.438^a^	0.672	0.17^d^
	No	1.72 ± 0.24				1.77 ± 0.29				1.68 ± 0.19			
Presence of relapse	Yes	1.78 ± 0.26	0.381^a^	0.514	0.12^d^	1.88 ± 0.26	0.716^a^	0.911	0.08^d^	1.72 ± 0.25	0.543^a^	0.735	0.11^d^
	No	1.75 ± 0.28				1.86 ± 0.30				1.69 ± 0.25			
Frequency of relapses	1	1.77 ± 0.29	0.650^c^	0.702	0.00^f^	1.96 ± 0.30	0.404^c^	0.594	0.06^f^	1.70 ± 0.25	0.402^c^	0.672	0.04^f^
	≥ 2	1.82 ± 0.23				1.83 ± 0.21				1.81 ± 0.27			
	≥ 3	1.75 ± 0.23				1.84 ± 0.28				1.68 ± 0.17			

*Note:* Normality was assessed using the Shapiro–Wilk test. Bold values indicate statistical significance (*p* < 0.05).

Abbreviations: AAN, atypical anorexia nervosa; AGR, albumin‐globulin ratio; AN, anorexia nervosa; AN‐R, restricting type anorexia nervosa; AN‐BP, binge eating‐purging type anorexia nervosa; TBW, target body weight.

^a^ Independent samples *t*‐test.

^b^ Mann–Whitney *U* test.

^c^ One way ANOVA test.

^d^ Cohen's *d*.

^e^ Effect size *r* (from Mann–Whitney *U* test).

^f^ Eta Squared (*η* ^2^) (from One way ANOVA test).

**TABLE 3:** The relationship between AGR and continuous variables.

	Total			AN			AAN		
	Correlation^a^	*p*	*q*	Correlation^a^	*p*	*q*	Correlation^a^	*p*	*q*
Disease severity									
Body weight	−0.152	**0.015**	0.055	−0.084	0.429	0.782	−0.044	0.571	0.802
BMI	−0.189	**0.002**	0.025	−0.107	0.316	0.726	−0.075	0.334	0.653
mBMI%	−0.174	**0.005**	0.030	0.007	0.945	0.978	−0.079	0.310	0.653
BMI *z*‐score	−0.176	**0.005**	0.030	−0.106	0.320	0.726	−0.033	0.677	0.802
Calorie intake at presentation	−0.041	0.557	0.730	0.071	0.542	0.800	−0.059	0.500	0.792
Duration of illness	0.022	0.738	0.854	0.009	0.935	0.978	0.019	0.817	0.907
Duration of weight loss	0.119	0.068	0.167	0.153	0.165	0.608	0.054	0.510	0.792
Amount of weight loss	0.034	0.609	0.781	0.081	0.463	0.782	0.014	0.863	0.921
Minimum body weight	−0.106	0.104	0.218	−0.040	0.717	0.929	0.037	0.654	0.802
Weight loss percentage	0.115	0.080	0.182	0.155	0.157	0.608	0.012	0.882	0.921
Weight loss rate	−0.006	0.922	0.954	−0.002	0.982	0.982	−0.011	0.890	0.921
Secondary amenorrhoea duration	0.146	0.164	0.302	0.141	0.302	0.726	−0.008	0.964	0.964
Hospitalisation length	−0.004	0.979	0.980	−0.080	0.724	0.929	0.085	0.680	0.802
Vital signs' recovery time	0.060	0.710	0.850	−0.102	0.686	0.920	0.216	0.323	0.653
Vital signs									
Supine systolic BP	−0.157	**0.012**	0.047	−0.023	0.827	0.977	−0.127	0.105	0.364
Supine diastolic BP	−0.158	**0.012**	0.047	−0.016	0.878	0.977	−0.152	0.052	0.236
Standing systolic BP	−0.160	**0.011**	0.047	0.032	0.766	0.962	−0.158	**0.043**	0.236
Standing diastolic BP	−0.085	0.176	0.310	0.075	0.482	0.782	−0.08	0.306	0.653
Orthostatic systolic BP change	0.041	0.520	0.714	0.010	0.926	0.978	0.061	0.436	0.757
Orthostatic diastolic BP change	−0.068	0.278	0.432	0.081	0.451	0.782	−0.164	**0.036**	0.236
Supine HR	−0.188	**0.003**	0.025	−0.195	0.067	0.395	−0.130	0.098	0.364
Standing HR	−0.192	**0.002**	0.025	−0.262	**0.013**	0.270	−0.106	0.174	0.489
HR change	−0.069	0.274	0.432	−0.120	0.259	0.726	−0.034	0.667	0.802
Body temperature	−0.039	0.538	0.721	−0.071	0.517	0.782	−0.023	0.776	0.881
Electrocardiogram findings									
Pulse rate	−0.096	0.179	0.310	−0.051	0.663	0.912	−0.068	0.452	0.762
QTc interval	0.016	0.819	0.879	−0.059	0.618	0.889	0.061	0.500	0.792
Laboratory data									
Haemoglobin	0.124	0.051	0.143	0.083	0.443	0.782	0.145	0.066	0.278
WBC	−0.207	**0.001**	0.025	−0.082	0.452	0.782	−0.213	**0.007**	0.207
ANC	−0.191	**0.003**	0.025	−0.117	0.282	0.726	−0.179	**0.023**	0.236
ALC	−0.079	0.218	0.357	0.074	0.494	0.782	−0.129	0.102	0.364
Platelet count	−0.199	**0.002**	0.025	−0.245	**0.022**	0.270	−0.162	**0.040**	0.236
Neutrophil‐lymphocyte ratio	−0.103	0.107	0.218	−0.112	0.303	0.726	−0.075	0.343	0.653
Platelet‐lymphocyte ratio	−0.029	0.646	0.794	−0.188	0.081	0.435	0.033	0.675	0.802
ALT	−0.009	0.894	0.942	0.218	**0.043**	0.308	−0.04	0.619	0.802
AST	0.020	0.759	0.855	0.110	0.311	0.726	−0.031	0.695	0.804
Total bilirubin	0.176	**0.009**	0.044	−0.022	0.844	0.977	0.260	**0.002**	0.118
Direct bilirubin	0.142	**0.034**	0.111	−0.004	0.972	0.982	0.206	**0.014**	0.221
BUN	0.019	0.768	0.855	0.020	0.849	0.977	−0.017	0.830	0.907
Creatinine	0.127	**0.046**	0.139	0.241	**0.026**	0.270	−0.007	0.933	0.949
BUN‐creatinine ratio	−0.051	0.427	0.752	−0.090	0.408	0.782	−0.009	0.910	0.923
Sodium	−0.054	0.390	0.548	−0.228	**0.032**	0.270	0.095	0.225	0.603
Phosphorus	−0.061	0.338	0.499	0.046	0.665	0.912	−0.088	0.266	0.628
Potassium	−0.084	0.184	0.310	−0.018	0.864	0.977	−0.042	0.595	0.802
Calcium	0.109	0.084	0.184	0.080	0.454	0.782	0.114	0.147	0.457
Magnesium	0.191	**0.005**	0.030	0.094	0.410	0.782	0.182	**0.033**	0.236
Glucose	−0.121	0.076	0.179	0.165	0.143	0.603	−0.169	0.051	0.236
Triglyceride	−0.075	0.385	0.548	0.012	0.930	0.978	−0.089	0.416	0.751
Total cholesterol	0.132	0.126	0.240	0.222	0.117	0.531	−0.055	0.618	0.802
HDL	0.274	**0.003**	0.025	0.342	**0.025**	0.270	0.115	0.342	0.653
LDL	0.140	0.113	0.222	0.243	0.092	0.452	−0.047	0.676	0.802
FSH	−0.296	**0.033**	0.111	−0.127	0.512	0.782	−0.440	**0.036**	0.236
LH	−0.384	**0.007**	0.038	−0.229	0.242	0.726	−0.537	**0.015**	0.221
Oestradiol	−0.295	**0.047**	0.139	−0.274	0.176	0.610	−0.318	0.171	0.489
TSH	0.131	0.061	0.159	0.230	**0.047**	0.308	0.130	0.141	0.457
fT3	−0.131	0.316	0.478	−0.451	**0.016**	0.270	0.112	0.536	0.802
fT4	−0.016	0.819	0.879	0.113	0.334	0.730	−0.073	0.420	0.751
Lumbar BMD *z*‐score	−0.049	0.637	0.794	0.196	0.186	0.610	−0.163	0.264	0.628
Femoral head BMD *z*‐score	0.003	0.980	0.980	0.339	**0.028**	0.270	−0.180	0.247	0.628
Prognosis									
Duration of the resumption of menstrual cycles	0.225	0.062	0.159	0.108	0.490	0.782	0.384	**0.048**	0.236
Time required to reach TBW	−0.038	0.720	0.850	−0.035	0.803	0.977	−0.087	0.616	0.802

*Note:* Bold values indicate statistical significance (*p* < 0.05).

Abbreviations: AAN, atypical anorexia nervosa; ALC, absolute lymphocyte count; ALT, alanine aminotransferase; AN, anorexia nervosa; ANC, absolute neutrophil count; AST, aspartate aminotransferase; BMD, bone mineral density; BMI, body mass index; BP, blood pressure; BUN, blood urea nitrogen; FSH, follicle stimulating hormone; fT3, free triiodothyronine; fT4, free thyroxine; HDL, high density lipoproteins; HR, heart rate; LDL, low density lipoproteins; LH, luteinising hormone; mBMI%, median BMI percent; TBW, target body weight; TSH, thyroid stimulating hormone; WBC, white blood cell.

^a^ Pearson correlation coefficient.

#### 3.2.1 Disease Severity

Among the parameters indicating the severity of the disease, AGR was found to be significantly higher in adolescents with an indication for hospitalisation at admission (1.86 ± 0.30 vs. 1.75 ± 0.30; *p* = 0.005) and in adolescents who have secondary amenorrhoea (1.80 ± 0.33 vs. 1.75 ± 0.29; *p* = 0.029). There was no significant association between AGR and presence of refeeding syndrome (*p* = 0.608; Table 2). According to comparisons between AGR groups, indication for hospitalisation at admission (13.0% vs. 28.4%; *p* = 0.002) and secondary amenorrhoea (36.4% vs. 55.6%; *p* = 0.005) were significantly higher in the high AGR group. There was no significant difference in the frequency of presence of refeeding syndrome between high and low AGR groups (*p* > 0.999; Table 4).

**TABLE 4:** Comparison of high and low AGR across categorical variables with effect sizes.

Characteristics	AGR < 1.84 (*n* = 161)	AGR ≥ 1.84 (*n* = 95)	*p*	*q*	Effect size
General characteristics					
Sex, *n* (%)			0.322^a^	0.616	0.06^c^
Female	144 (89.4%)	81 (85.3%)			
Male	17 (10.6%)	14 (14.7%)			
ED‐subtype, *n* (%)			**<** **0.001** ^a^	0.015	0.25^c^
AN	42 (26.1%)	48 (50.5%)			
AAN	119 (73.9%)	47 (49.5%)			
AN‐subtype, *n* (%)			0.087^a^	0.255	0.11^c^
AN‐R	117 (72.7%)	78 (82.1%)			
AN‐BP	44 (27.3%)	17 (17.9%)			
Disease severity					
Secondary amenorrhoea, *n* (%)	52 (36.4%)	45 (55.6%)	**0.005** ^a^	0.044	0.19^c^
Indication for hospitalisation at admission, *n* (%)	21 (13.0%)	27 (28.4%)	**0.002** ^a^	0.022	0.19^c^
Presence of refeeding syndrome, *n* (%)	8 (5.1%)	4 (4.3%)	> 0.999^b^	0.999	0.02^c^
Vital sign instabilities, *n* (%)					
Hypotension, systolic BP < 90 mmHg	21 (13.0%)	22 (23.4%)	**0.033** ^a^	0.145	0.13^c^
Orthostatic systolic BP change ≥ 10 mmHg	64 (39.8%)	36 (38.7%)	0.870^a^	0.999	0.01^c^
Bradycardia, < 50 beats per min	3 (1.9%)	7 (7.5%)	**0.041** ^b^	0.150	0.14^c^
Orthostatic HR change, ≥ 20 beats per min	71 (44.4%)	37 (39.4%)	0.435^a^	0.684	0.05^c^
Hypothermia, < 36.0°C	9 (5.7%)	12 (13.3%)	**0.039** ^a^	0.150	0.13^c^
Laboratory abnormalities, *n* (%)					
Leukopenia, < 1.5 × 10^3^/µL	4 (2.6%)	10 (10.8%)	**0.007** ^a^	0.051	0.17^c^
Lymphopenia, < 1 × 10^3^/µL	2 (1.3%)	2 (2.2%)	0.632^b^	0.898	0.03^c^
Thrombocytopaenia, < 150 × 10^3^/µL	0 (0.0%)	1 (1.1%)	0.377^b^	0.638	0.08^c^
High ALT, > 40 U/L	147 (95.5%)	83 (90.2%)	0.107^a^	0.262	0.10^c^
High AST, > 40 U/L	145 (94.2%)	86 (94.5%)	0.909^a^	0.999	0.01^c^
Hyperbilirubinemia, > 1.2 mg/dL	11 (8.0%)	16 (18.8%)	**0.016** ^a^	0.094	0.16^c^
High BUN, > 20 mg/dL	7 (4.5%)	7 (7.4%)	0.318^a^	0.616	0.06^c^
High creatinine, > 0.8 mg/dL	8 (5.2%)	10 (10.8%)	0.100^a^	0.262	0.10^c^
Hyponatremia, < 135 mEq/L	3 (1.9%)	4 (4.3%)	0.429^b^	0.684	0.07^c^
Hypophosphatemia, ≤ 2.9 mg/dL	3 (2.0%)	0 (0.0%)	0.289^b^	0.616	0.09^c^
Hypokalemia, ≤ 3.5 mEq/L	6 (3.8%)	2 (2.1%)	0.714^b^	0.917	0.05^c^
Hypocalcemia, < 8.5 mg/dL	1 (0.6%)	0 (0.0%)	> 0.999^b^	0.999	0.05^c^
Hypomagnesaemia, ≤ 1.7 mg/dL	4 (2.9%)	1 (1.2%)	0.653^b^	0.898	0.05^c^
Hypoglycemia, < 60 mg/dL	6 (4.5%)	5 (6.1%)	0.752^b^	0.919	0.03^c^
Hypertriglyceridemia, ≥ 130 mg/dL	4 (5.1%)	3 (5.2%)	> 0.999^b^	0.999	0.00^c^
High triglyceride, ≥ 90 mg/dL	22 (27.8%)	18 (31.0%)	0.685^a^	0.913	0.03^c^
Hypercholesterolaemia, ≥ 200 mg/dL	21 (26.6%)	24 (42.1%)	0.058^a^	0.196	0.16^c^
High total cholesterol, ≥ 170 mg/dL	42 (53.2%)	36 (63.2%)	0.245^a^	0.567	0.10^c^
Low HDL, ≤ 45 mg/dL	0 (0.0%)	0 (0.0%)			
High LDL, ≥ 130 mg/dL	69 (87.3%)	50 (87.7%)	0.948^a^	0.999	0.01^c^
Moderately high LDL, ≥ 110 mg/dL	41 (53.9%)	33 (62.3%)	0.347^a^	0.636	0.08^c^
Low FSH, < 2 mIU/mL	5 (17.9%)	11 (45.8%)	**0.029** ^a^	0.142	0.30^c^
Low LH, < 2 mIU/mL	12 (46.2%)	20 (90.9%)	**0.001** ^a^	0.015	0.47^c^
Low oestradiol, < 20 pg/mL	9 (36.0%)	15 (71.4%)	**0.017** ^a^	0.094	0.35^c^
Low fT3, < 3.5 pmol/L	0 (0.0%)	1 (4.3%)	0.377^b^	0.638	0.17^c^
Low lumbar BMD *z*‐score, ≤ −2 SD	3 (6.7%)	6 (11.8%)	0.495^b^	0.751	0.09^c^
Low femoral head BMD *z*‐score, ≤ −2 SD	5 (12.5%)	4 (8.9%)	0.729^b^	0.917	0.06^c^
Prognosis					
Resumption of menstrual cycles, *n* (%)	35 (89.7%)	36 (92.3%)	> 0.999^b^	0.999	0.04^c^
Achievement of TBW, *n* (%)	42 (71.2%)	49 (84.5%)	0.084^a^	0.255	0.16^c^
Presence of hospitalisation during follow‐up, *n* (%)	24 (18.9%)	35 (45.5%)	**<** **0.001** ^a^	0.015	0.28^c^
Frequency of additional hospitalisations, *n* (%)			> 0.999^b^	0.999	0.02^c^
1	28 (90.3%)	33 (89.2%)			
≥ 2	3 (9.7%)	4 (10.8%)			
Presence of remission, *n* (%)	96 (75.6%)	63 (81.8%)	0.298^a^	0.616	0.07^c^
Presence of relapse, *n* (%)	47 (34.6%)	37 (45.7%)	0.104^a^	0.262	0.11^c^
Frequency of relapses, *n* (%)			0.641^a^	0.898	0.05^c^
1	23 (48.9%)	20 (54.1%)			
≥ 2	24 (51.1%)	17 (45.9%)			

*Note:* Bold values indicate statistical significance (*p* < 0.05).

Abbreviations: AAN, atypical anorexia nervosa; AGR, albumin‐globulin ratio; ALT, alanine aminotransferase; AN, anorexia nervosa; AN‐BP, binge eating‐purging type anorexia nervosa; AN‐R, restricting type anorexia nervosa; AST, aspartate aminotransferase; BMD, bone mineral density; BP, blood pressure; BUN, blood urea nitrogen; ED, eating disorder; FSH, follicle stimulating hormone; fT3, free triiodothyronine; HDL, high density lipoproteins; HR, heart rate; LDL, low density lipoproteins; LH, luteinising hormone; TBW, target body weight.

^a^ Pearson's Chi‐square test.

^b^ Fisher's exact *t* Test.

^c^ Cramer's *V* (for categorical variables).

Among participants significant negative correlations were found between AGR and body weight (*p* = 0.015, *r* = −0.152), BMI (*p* = 0.002, *r* = −0.189), mBMI% (*p* = 0.005, *r* = −0.174) and BMI *z*‐score (*p* = 0.005, *r* = −0.176; Table 3). Body weight (49.80 ± 11.53 vs. 45.95 ± 11.10 kg; *p* = 0.002), BMI (18.87 ± 3.62 vs. 17.44 ± 3.27 kg/m^2^; *p* = 0.001), mBMI% (93.77 ± 18.11 vs. 86.29 ± 14.35%; *p* = 0.001) and BMI *z*‐score (−0.81 ± 1.74 vs. −1.47 ± 1.72; *p* = 0.001) also showed significant differences between the AGR groups, with all of these values being significantly lower in the high AGR group (Table 5).

**TABLE 5:** Comparison of high and low AGR across continuous variables with effect sizes.

Characteristics	AGR < 1.84 (*n* = 161)	AGR ≥ 1.84 (*n* = 95)	*p*	*q*	Effect size
General characteristics, mean ± SD					
Age, years	15.09 ± 1.53	15.06 ± 1.52	0.955^b^	0.988	0.00^d^
Disease severity, mean ± SD					
Body weight, kg	49.80 ± 11.53	45.95 ± 11.10	**0.002** ^b^	0.013	0.19^d^
BMI, kg/m^2^	18.87 ± 3.62	17.44 ± 3.27	**0.001** ^b^	0.009	0.20^d^
mBMI%, %	93.77 ± 18.11	86.29 ± 14.35	**0.001** ^b^	0.009	0.22^d^
BMI *z*‐score	−0.81 ± 1.74	−1.47 ± 1.72	**0.001** ^b^	0.009	0.21^d^
Calorie intake at presentation, kcal	868.60 ± 377.14	798.43 ± 365.35	0.216^a^	0.341	0.19^c^
Duration of illness, months	9.65 ± 9.16	11.63 ± 11.14	**0.025** ^b^	0.068	0.14^d^
Duration of weight loss, months	5.42 ± 4.60	6.82 ± 5.98	0.091^b^	0.182	0.11^d^
Amount of weight loss, kg	13.90 ± 9.16	14.76 ± 8.35	0.274^b^	0.401	0.07^d^
Minimum body weight, kg	46.74 ± 10.49	45.01 ± 11.64	0.085^b^	0.176	0.11^d^
Maximum body weight, kg	61.30 ± 14.93	59.67 ± 14.58	0.389^b^	0.519	0.06^d^
Weight loss percentage, %	21.47 ± 10.00	24.58 ± 10.52	**0.017** ^b^	0.051	0.16^d^
Weight loss rate, %/months	6.01 ± 3.79	6.43 ± 6.36	0.471^b^	0.612	0.05^d^
Secondary amenorrhoea duration, months	5.14 ± 2.68	7.50 ± 5.80	**0.044** ^b^	0.106	0.21^d^
Hospitalisation length, days	27.55 ± 45.34	19.96 ± 11.96	0.756^b^	0.856	0.05^d^
Vital signs recovery time, days	8.88 ± 8.41	9.00 ± 6.69	0.623^b^	0.761	0.08^d^
Vital signs					
Supine systolic BP, mmHg	100.40 ± 12.84	95.37 ± 12.23	**0.001** ^b^	0.009	0.20^d^
Supine diastolic BP, mmHg	61.27 ± 8.26	58.62 ± 8.87	**0.017** ^b^	0.051	0.14^d^
Standing systolic BP, mmHg	97.80 ± 14.22	92.74 ± 13.30	**0.004** ^b^	0.022	0.18^d^
Standing diastolic BP, mmHg	61.71 ± 10.59	59.41 ± 9.72	0.118^b^	0.215	0.10^d^
Orthostatic systolic BP change, mmHg	6.33 ± 6.14	5.97 ± 5.33	0.905^b^	0.987	0.01^d^
Orthostatic diastolic BP change, mmHg	6.16 ± 5.39	5.16 ± 4.92	0.192^b^	0.320	0.08^d^
Supine HR, beats/min	73.14 ± 14.79	69.29 ± 14.59	0.054^b^	0.125	0.12^d^
Standing HR, beats/min	92.60 ± 18.95	84.66 ± 18.04	**0.001** ^b^	0.009	0.21^d^
HR change, beats/min	19.84 ± 12.61	16.38 ± 11.46	**0.027** ^b^	0.070	0.14^d^
Body temperature, °C	36.65 ± 2.71	36.32 ± 0.60	**0.015** ^b^	0.051	0.15^d^
Electrocardiogram findings					
Pulse rate	69.62 ± 16.79	66.06 ± 14.49	0.114^b^	0.214	0.11^d^
Laboratory data					
Haemoglobin, g/dL	13.43 ± 1.19	13.76 ± 1.26	0.060^b^	0.133	0.12^d^
WBC, × 10^3^/µL	6.25 ± 1.87	5.58 ± 1.83	**0.007** ^b^	0.032	0.17^d^
ANC, × 10^3^/µL	3.56 ± 1.55	3.04 ± 1.43	**0.006** ^b^	0.030	0.18^d^
ALC, × 10^3^/µL	2.11 ± 0.68	2.04 ± 0.65	0.528^b^	0.660	0.04^d^
Platelet count, × 10^3^/µL	264.75 ± 63.80	243.76 ± 57.27	**0.016** ^b^	0.051	0.15^d^
Neutrophil‐lymphocyte ratio	1.86 ± 1.19	1.59 ± 0.94	**0.023** ^b^	0.066	0.14^d^
Platelet‐lymphocyte ratio	135.26 ± 46.11	129.57 ± 45.14	0.383^b^	0.519	0.06^d^
ALT, U/L	16.89 ± 18.23	18.15 ± 20.41	0.381^bb^	0.519	0.06^d^
AST, U/L	20.88 ± 11.36	22.74 ± 14.96	0.109^b^	0.211	0.10^d^
Total bilirubin, mg/dL	0.71 ± 0.42	0.93 ± 0.67	**0.013** ^b^	0.051	0.17^d^
Direct bilirubin, mg/dL	0.14 ± 0.07	0.18 ± 0.10	**0.011** ^b^	0.047	0.17^d^
BUN, mg/dL	11.34 ± 4.17	11.63 ± 5.50	0.857^b^	0.952	0.01^d^
Creatinine, mg/dL	0.62 ± 0.12	0.65 ± 0.13	0.173^b^	0.297	0.09^d^
Sodium, mEq/L	139.40 ± 2.03	139.29 ± 2.62	0.685^b^	0.806	0.03^d^
Phosphorus, mg/dL	3.91 ± 0.53	3.88 ± 0.52	0.479^b^	0.612	0.04^d^
Potassium, mEq/L	4.31 ± 0.45	4.28 ± 0.47	0.163^b^	0.288	0.09^d^
Calcium, mg/dL	9.94 ± 0.40	10.02 ± 0.49	0.239^b^	0.368	0.07^d^
Magnesium, mg/dL	2.09 ± 0.17	2.16 ± 0.19	**0.004** ^b^	0.022	0.20^d^
Glucose, mg/dL	81.46 ± 35.94	76.22 ± 12.32	0.079^b^	0.169	0.12^d^
Triglyceride, mg/dL	77.73 ± 30.26	74.72 ± 26.65	0.741^b^	0.855	0.03^d^
Total cholesterol, mg/dL	188.54 ± 67.42	197.40 ± 72.06	0.251^b^	0.377	0.10^d^
HDL, mg/dL	58.48 ± 14.37	64.82 ± 16.49	**0.031** ^b^	0.078	0.20^d^
LDL, mg/dL	119.24 ± 50.26	125.96 ± 55.84	0.281^b^	0.401	0.10^d^
FSH, mIU/mL	4.73 ± 2.40	2.67 ± 2.08	**0.002** ^b^	0.013	0.43^d^
LH, mIU/mL	3.23 ± 3.55	0.57 ± 1.22	**<** **0.001** ^b^	0.009	0.53^d^
Oestradiol, pg/mL	39.68 ± 39.29	15.73 ± 8.78	**0.001** ^b^	0.009	0.47^d^
TSH, mIU/mL	1.60 ± 0.82	1.79 ± 1.24	0.944^b^	0.988	0.01^d^
fT3, pmol/L	4.99 ± 1.04	4.67 ± 0.89	0.216^a^	0.341	0.33^c^
fT4, pmol/L	10.59 ± 1.92	10.60 ± 1.86	> 0.999^b^	0.999	0.00^d^
Lumbar BMD *z*‐score, g/m^2^	−0.40 ± 1.28	−0.55 ± 1.02	0.980^b^	0.997	0.00^d^
Femoral head BMD *z*‐score, g/m^2^	−0.54 ± 1.43	−0.72 ± 0.99	0.634^b^	0.761	0.05^d^
Prognosis, mean ± SD					
Duration of the resumption of menstrual cycles, months	5.53 ± 4.32	9.35 ± 8.29	**0.016** ^b^	0.051	0.29^d^
Time required to reach TBW, months	4.57 ± 5.14	4.82 ± 5.54	0.945^b^	0.988	0.01^d^

*Note:* Normality was assessed using the Shapiro–Wilk test. Bold values indicate statistical significance (*p* < 0.05).

Abbreviations: AGR, albumin‐globulin ratio; ALC, absolute lymphocyte count; ALT, alanine aminotransferase; ANC, absolute neutrophil count; AST, aspartate aminotransferase; BMD, bone mineral density; BMI, body mass index; BP, blood pressure; BUN, blood urea nitrogen; FSH, follicle stimulating hormone; fT3, free triiodothyronine; fT4, free thyroxine; HDL, high density lipoproteins; HR, heart rate; LDL, low density lipoproteins; LH, luteinising hormone; mBMI%, median BMI percent; TBW, target body weight; TSH, thyroid stimulating hormone; WBC, white blood cell.

^a^ Independent samples *t*‐test.

^b^ Mann–Whitney *U* test.

^c^ Cohen's *d*.

^d^ Effect size *r* (from Mann–Whitney *U* test).

When the relationship between vital signs and AGR was evaluated, supine systolic (*p* = 0.012, *r* = −0.157) and diastolic blood pressure (*p* = 0.012, *r* = −0.158), standing systolic blood pressure (*p* = 0.011, *r* = −0.160) and supine (*p* = 0.003, *r* = −0.188) and standing heart rate (*p* = 0.002, *r* = −0.192) showed significant negative correlations in all patients (Table 3). Regarding vital sign instabilities; hypotension (13% vs. 23.4%; *p* = 0.033), bradycardia (1.9% vs. 7.5%; *p* = 0.041), and hypothermia (5.7% vs. 13.3%; *p* = 0.039) occurred significantly more frequent in the high AGR group. There was no difference in the frequency of orthostatic systolic blood pressure or heart rate changes between the two groups (*p* = 0.870 and *p* = 0.435, respectively; Table 4).

According to laboratory data in all groups, a significant positive correlation was found between AGR and total bilirubin (*p* = 0.009, *r* = 0.176), direct bilirubin (*p* = 0.034, *r* = 0.142), creatinine (*p* = 0.046, *r* = 0.127), magnesium (*p* = 0.005, *r* = 0.191), and HDL (*p* = 0.003, *r* = 0.274). However, there was a significant negative correlation between AGR and WBC (*p* = 0.001, *r* = −0.207), ANC (*p* = 0.003, *r* = −0.191), platelet count (*p* = 0.002, *r* = −0.199), FSH (*p* = 0.033, *r* = −0.296), LH (*p* = 0.007, *r* = −0.384) and oestradiol (*p* = 0.047, *r* = −0.295). Although there was no significant correlation between AGR and lumbar and femoral head BMD *z*‐scores in all groups (*p* = 0.637 and *p* = 0.980, respectively), femoral head BMD *z*‐score showed significant positive correlation with AGR in AN group (*p* = 0.028, *r* = 0.339; Table 3). In terms of laboratory abnormalities, leucopenia (2.6% vs. 10.8%; *p* = 0.007), hyperbilirubinemia (8.0% vs. 18.8%; *p* = 0.016), low FSH (17.9% vs. 45.8%; *p* = 0.029), low LH (46.2% vs. 90.9%; *p* = 0.001), and low oestradiol (36.0% vs. 71.4%; *p* = 0.017) were significantly more frequent in the high AGR group. The frequencies of low lumbar and femoral head BMD *z*‐scores did not differ significantly between the two groups (*p* = 0.495 and *p* = 0.729, respectively; Table 4).

#### 3.2.2 Prognosis

Among the parameters indicating prognosis, AGR was found to be significantly higher in adolescents who required additional hospitalisation during follow‐up than in those who did not in all groups (1.90 ± 0.27 vs. 1.71 ± 0.24; *p* < 0.001) and in AAN group (1.86 ± 0.24 vs. 1.67 ± 0.22; *p* < 0.001). However, there was no significant association correlation between AGR and resumption of menstrual cycles (*p* = 0.370), achievement of TBW (*p* = 0.261), presence of remission (*p* = 0.184) and relapses (*p* = 0.381; Table 2). There was a significant positive correlation between AGR and duration of the resumption of menstrual cycles in AAN group (*p* = 0.048, *r* = 0.384). Although there was also a positive correlation between AGR and duration of the resumption of menstrual cycles in all patients (*p* = 0.062, *r* = 0.225) and in AN group (*p* = 0.490, *r* = 0.108), this correlation was not significant. When prognosis data were reviewed between AGR groups, the frequency of hospitalisation during follow‐up was found to be significantly higher in the high AGR group (18.9% vs. 45.5%; *p* < 0.001), while the frequency of the resumption of menstrual cycles (*p* > 0.999), achievement of TBW (*p* = 0.084), presence of remission (*p* = 0.298) and relapse (*p* = 0.104) did not differ between the two groups (Table 4). The duration of the resumption of menstrual cycles was significantly longer in the high AGR group (5.53 ± 4.32 vs. 9.35 ± 8.2 months; *p* = 0.016; Table 5).

## 4 Discussion

To the best of our knowledge, this is the first study to evaluate AGR as a biochemical marker of disease severity and prognosis in adolescents with AN and AAN. Our most important finding is that higher AGR values at presentation was associated with indicators of greater medical disease severity and poorer prognosis in terms of increased need for rehospitalisation during follow‐up and longer duration of the resumption of menstrual cycles. Among disease severity markers higher AGR values most significantly associated with need for hospitalisation at presentation ‐particularly vital sign abnormalities‐, and secondary amenorrhoea. These findings suggest that AGR as a simple and inexpensive parameter, can assist in risk assessment and treatment planning.

Although albumin is widely regarded as a marker of malnutrition and low AGR has been associated with poorer outcomes, we found that higher AGR was associated with a more severe medical course in AN. Unlike in severe protein‐energy malnutrition such as kwashiorkor (Coulthard 2015), patients with AN often exhibit preserved hepatic synthetic function, minimal systemic inflammation, and compensatory metabolic adaptations, all of which may contribute to serum albumin levels remaining within normal limits (Mehler and Koutsavlis 2021). On the other hand, Seitz et al. reported that serum immunoglobulin G (IgG) levels and α‐melanocyte‐stimulating hormone (α‐MSH) reactive IgG production were directly proportional to BMI z‐scores in patients with AN and increased during 1‐year follow‐up (Seitz et al. 2024). Roubalova et al. (2021) documented significantly reduced α1‐, β‐ and γ‐globulin fractions at admission, which returned to normal after nutritional rehabilitation, supporting a direct link between globulin depletion and disease activity. However, studies have shown that serum albumin often remains within the reference range even in extremely low weights (Krantz et al. 2005; Narayanan et al. 2009). This may be explained by the fact that albumin is exclusively synthesised in the liver, whereas globulins include both liver‐synthesised types, such as α‐ and β‐globulins, and immune‐related γ‐globulins produced by plasma cells. Albumin also has a slower turnover and may be preferentially preserved by the liver during malnutrition. It was also demonstrated that albumin, irrespective of nutritional status, is a negative inflammatory phase marker (Mirsaeidi et al. 2018). When these physiological differences are considered, higher AGR resulting from relatively preserved albumin in the face of low globulin may reflect the disease severity.

In our study, we found significant associations between AGR and both clinical and laboratory indicators of disease severity. Most notably, the need for hospitalisation and vital sign instabilities such as hypotension, bradycardia and hypothermia was associated with high AGR levels, suggesting that higher AGR values may be reflective of more advanced physiological compromise and could serve as a useful marker for identifying patients requiring more intensive clinical care. We also observed a parallel decrease in WBC, ANC and platelet counts with the increase in AGR and more leucopenia in the high AGR group. Normocytic anaemia, leucopenia and thrombocytopaenia occur in approximately one‐third of severely malnourished AN cases and almost half exhibit atrophic or ‘gelatinous’ bone marrow transformation (Boullu‐Ciocca et al. 2005; Hütter et al. 2009). Studies have confirmed that these cytopenias correlate with disease severity and generally improve after nutritional rehabilitation (Cleary et al. 2010; Sabel et al. 2013). In our study, there was a positive correlation between AGR and creatinine. Downey et al. (2022) identified marked renal dysfunction at presentation as an indicator of disease severity in patients with AN and AAN. Importantly, the elevation of AGR in our study appears independent of dehydration. Despite a positive correlation with creatinine, it did not correlate with BUN‐creatinine ratio, which is a more specific index of volume depletion, and it correlated negatively with heart rate, whereas dehydration typically produces increased heart rate. These findings support that AGR may enhance risk stratification in severe AN and AAN cases by holistically capturing not only nutritional depletion but also concurrent haematopoietic, nephrotic and immune deterioration.

We observed that the high AGR group showed more frequent secondary amenorrhoea and low FSH, LH, and oestradiol levels. Amenorrhoea due to hypogonadotropic hypogonadism is a well‐recognised hallmark of profound energy deficiency and neuroendocrine adaptation in AN and AAN (Haines 2023; Misra and Klibanski 2014). Suppression of gonadotrophins and oestradiol closely tracks caloric deficit and reverses only after substantial nutritional rehabilitation, generally occurring once patients regain 90% or above of median body weight (Golden et al. 2008; Misra and Klibanski 2014). The association we found between AGR and hypothalamic‐pituitary‐gonadal suppression in our study reinforce the feature of AGR as a marker that captures the medical severity.

In our study, we found a negative correlation between BMI *z*‐scores and AGR values. Similarly, high AGR group had lower BMI *z*‐scores and adolescents with AN had higher AGR levels than AAN. However, this association was not observed for the duration of illness and weight loss rate. These findings suggest that body composition, specifically muscle and adipose tissue mass, should be considered when interpreting AGR outcomes, as both contribute to protein metabolism and systemic inflammation. A previous study in adults showed a sex‐based association, that while albumin was positively correlated with muscle mass in men, the association was negative in women (Chen et al. 2022). For globulin and muscle mass while a negative association was observed in men, the association was nonspecific in women. Since AN primarily affects women and adolescents with AAN have been shown to have higher fat mass and lean body mass index, these findings could explain the negative association between body weight and AGR. Another possible explanation is that changes in AGR may follow a non‐linear pattern throughout the course of AN in relation to disease severity, which should be considered when interpreting its associations with clinical outcomes.

The positive association of higher AGR with rehospitalisation at follow‐up and longer duration of the resumption of menstrual cycles underscores its prognostic value, whereas the lack of correlation with resumption of menstrual cycles, achievement of TBW or remission suggests that additional medical and psychosocial factors that may affect long‐term outcomes (Gorrell et al. 2023; Lau et al. 2024; Norrlin and Baumann 2025).

Limitations include the relatively small sample size, and retrospective, single‐centre design, which may restrict generalisability. The absence of serial AGR measurements prevents reassessment after nutritional rehabilitation. Serum protein electrophoresis was not performed in our study, so specific globulin fractions could not be distinguished, and protein concentrations may have been influenced by hydration status, which we did not systematically assess. In addition, previous growth charts were missing in some AN patients, and the retrospective design did not allow for reassessment of missing growth curves. Therefore, we used 90% of IBW as an approximate threshold for AN patients, which may not fully reflect each patient's growth potential and could result in under‐ or overestimation of target weight status. Finally, due to the retrospective nature of this study, psychological remission with validated tools and other predictors of long‐term outcomes such as psychosocial and treatment factors, could not be assessed. However, as the study focused on physiological outcomes, this limitation does not substantially impact the main results.

In conclusion, our data show that higher AGR is a significant predictor of greater medical severity and associated with future hospitalisation risk. AGR is inexpensive and a part of standard chemistry panels. Prospective, multicenter studies should explore AGR changes with serial measurements after nutritional rehabilitation and evaluate composite indices combining AGR with haematologic and inflammatory biomarkers to personalise care for youth with restrictive eating disorders.

## Author Contributions

**Eylem Şerife Kalkan:** conceptualization, methodology, project administration, writing – original draft, writing – review and editing. **Ayşe Bilge Baklaci:** conceptualization, methodology, writing – original draft. **Yelda Kiliç:** conceptualization, methodology, writing – original draft. **Sinem Akgül:** conceptualization, methodology, supervision, writing – original draft, writing – review and editing. **Nilgün Özgül:** conceptualization, methodology, formal analysis. **Melis Pehlivanturk Kizilkan:** conceptualization, methodology, project administration, supervision, writing – original draft, writing – review and editing.

## Funding

The authors have nothing to report.

## Ethics Statement

The study protocol was approved by the Health Sciences Research Ethics Committee of Hacettepe University (decision no: 2024/21‐12).

## Conflicts of Interest

The authors declare conflicts to declare.

## Supporting information


**Table S1:** The cut‐off values for laboratory data.

## Data Availability

Data will be available upon request to the corresponding author.
