# Supplementary material for: Assessing Clinical Severity and Prognosis in Adolescents With Anorexia Nervosa and Atypical Anorexia Nervosa Using the Albumin‐Globulin Ratio
Source: Eur Eat Disord Rev. 2026 Apr 4;34(5):1193–208. doi: 10.1002/erv.70106 (PMC13432581; doi:10.1002/erv.70106)
Supplement: Supplementary file 1 — Table S1: The cut‐off values for laboratory data. [file ERV-34-1193-s001.docx]

**Table S1.** The cut-off values for laboratory data

| Leukopenia | <1.5x10^3^/µL |
| --- | --- |
| Lymphopenia | <1x10^3^/µL |
| Thrombocytopenia | <150x10^3^/µL |
| High ALT | >40 U/L |
| High AST | >40 U/L |
| Hyperbilirubinemia | >1.2 mg/dL |
| High BUN | >20 mg/dL |
| High creatinine | >0.8 mg/dL |
| Hyponatremia | <135 mEq/L |
| Hypophosphatemia | ≤2.9 mg/dL |
| Hypokalemia | ≤3.5 mEq/L |
| Hypocalcemia | <8.5 mg/dL |
| Hypomagnesemia | ≤1.7 mg/dL |
| Hypoglycemia | <60 mg/dL |
| Hypertriglyceridemia | ≥130 mg/dL |
| High triglyceride | ≥90 mg/dL |
| Hypercholesterolemia | ≥200 mg/dL |
| High total cholesterol | ≥170 mg/dL |
| Low HDL | ≤45 mg/dL |
| High LDL | ≥130 mg/dL |
| Moderately high LDL | ≥110 mg/dL |
| Low FSH | <2 mIU/mL |
| Low LH | <2 mIU/mL |
| Low estradiol | <20 pg/mL |
| Low fT3 | <3.5 pmol/L |

*ALT: alanine aminotransferase, AST: aspartate aminotransferase, BUN: blood urea nitrogen, HDL: high density lipoproteins, LDL: low density lipoproteins, FSH: follicle stimulating hormone, LH: luteinizing hormone, fT3: free triiodothyronine*
